# Transcriptional and functional characterization in the terpenoid precursor pathway of the early land plant *Physcomitrium patens*

**DOI:** 10.1111/plb.13741

**Published:** 2024-11-27

**Authors:** A. Horn, Y. Lu, F. J. Astorga Ríos, H. Toft Simonsen, J. D. Becker

**Affiliations:** ^1^ ITQB NOVA—Instituto de Tecnologia Química e Biológica António Xavier Oeiras Portugal; ^2^ Instituto Gulbenkian de Ciência Oeiras Portugal; ^3^ Department of Biotechnology and Biomedicine Technical University of Denmark Kongens Lyngby Denmark; ^4^ Université Jean Monnet Saint‐Etienne, CNRS, LBVpam Saint‐Etienne France

**Keywords:** 1‐deoxy‐D‐xylulose‐5‐phosphate synthase, abiotic stress, DXS, isoprenoids, metabolomics, transcriptomics

## Abstract

- Isoprenoids comprise the largest group of plant specialized metabolites. 1‐deoxy‐D‐xylulose‐5‐phosphate synthase (DXS) is one of the major rate‐limiting enzymes in their biosynthesis. The DXS family expanded structurally and functionally during evolution and is believed to have significantly contributed to metabolic complexity and diversity in plants. This family has not yet been studied in *Physcomitrium patens* or other bryophytes.
- Here, we assessed the degree of evolutionary expansion in the DXS family in bryophytes and, more specifically, in *P. patens* using phylogenetic analysis. Transcriptome profiling was applied to investigate tissue‐specific, developmental, and environmental responses, such as salt stress, in the DXS family. Moreover, the effect of salt stress on terpenoid biosynthesis was monitored through metabolomics.
- The phylogenetic analysis of DXS revealed that a structural expansion occurred in bryophytes, but not in *P. patens*. Functional complementation assay revealed functional activity in all four copies. Comparative transcriptomics showed tissue‐ and condition‐specific divergence in the expression profiles of *DXS* copies and demonstrated specific stress responses for *PpDXS1D*, particularly to salt stress. These findings coincide with increased flux in the pathway towards downstream metabolites under salt stress. Additionally, co‐expression network analysis revealed significant differences between the co‐expressed genes of the DXS copies and illustrated enrichment of stress‐responsive genes in the *PpDXS1D* network.
- These results suggest that the DXS family in *P. patens* is conserved but undergoes differential transcriptional regulation, which might allow *P. patens* to fine‐tune DXS levels under different conditions, such as abiotic stress.

## Graphical abstract

Activity, and regulation of the 1‐deoxy‐D‐xylulose‐5‐phosphate synthase (DXS) gene family shows structural expansion in bryophytes and functional conservation in *Physcomitrium patens*, while DXS copies are differentially regulated under different conditions, including salt stress.

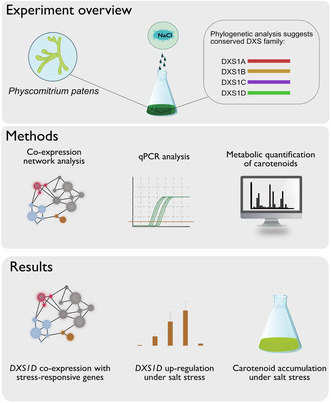

## INTRODUCTION

Isoprenoids (or terpenoids) comprise a wide group of structurally and functionally diverse metabolites, inherent to all living things. Amongst all the phyla, plants offer the highest diversity, with over 40000 identified compounds (Tholl 2015), some of which participate in essential processes, such as photosynthesis, electron transfer, and developmental regulation. The majority are related to specialized metabolisms, *e.g*., shaping plant–environment interactions, such as biotic stress, defence, and pollinator attraction (Pichersky & Raguso 2018). Peculiar characteristics, including their pleasant smell, colour, and antimicrobial properties, have also made them highly attractive for biotechnological application (Caputi & Aprea 2011; Rubulotta & Quadrelli 2019; Horn *et al*. 2021).

Isoprenoids are synthesized via two pathways, the mevalonate pathway in the cytosol, where hydroxymethylglutaryl‐CoA reductase (HMGR) is presumably rate‐limiting (Chappell *et al*. 1995), and via the methylerythritol 4‐phosphate (MEP) pathway in the chloroplasts, where the rate‐limitation is coordinated by 1‐deoxy‐D‐xylulose‐5‐phosphate synthase (DXS) (Estevez *et al*. 2001; Wright *et al*. 2014), which catalyses the condensation of pyruvate and D‐glyceraldehyde‐3‐phosphate into 1‐deoxy‐dexylulose‐5‐phosphate (DXP) (Lois *et al*. 2000; Querol *et al*. 2002). Both pathways result in synthesis of the direct isoprenoid precursors, isopentenyl diphosphate (IPP) and its isomer dimethylallyl diphosphate (DMAPP).

In plants, the DXS family can be phylogenetically divided into three clades with specialized functions (Cordoba *et al*. 2009; De Luna‐Valdez *et al*. 2021). Clade 1 DXS has been primarily associated with housekeeping functions, such as photosynthesis and developmental regulation in chloroplasts (Estévez *et al*. 2000; Carretero‐Paulet *et al*. 2013). Increased DXS1 levels trigger the accumulation of downstream products, such as pigments (carotenoids and chlorophylls), plant hormones (*e.g*., abscisic acid), and monoterpenes (Estevez *et al*. 2001; Munoz‐Bertomeu *et al*. 2006).

Clade 2 DXS enzymes are mostly located in non‐photosynthetic active plastids and specialized tissues, such as trichomes and roots (Walter *et al*. 2002; Hofberger *et al*. 2015; Zhang *et al*. 2018, 2020). These have been particularly linked to specialized metabolism. For example, in rice, OsDXS2 functions as a regulator in the carotenoid metabolism of seeds (You *et al*. 2020). Moreover, DXS type 2 has been associated with response to abiotic and biotic stress, *e.g*. in *Artemisia annua* (Zhang *et al*. 2018) and *Morus notabilis* (Zhang *et al*. 2020), although stress sensitivity has also been reported for *DXS1*, *e.g*., in *Pinus massonia* (Li *et al*. 2021). Similarly, DXS confer resistance to stress in poplar (Wei *et al*. 2019).

The function of clade 3 DXS is still debated. Because of its comparatively low expression, a role in the synthesis of terpenoid derivates that naturally occur at low concentrations, such as phytohormones, was discussed (Cordoba *et al*. 2011). Most recently, this hypothesis was challenged, as co‐expression network analysis of *AtDXS3* suggested neofunctionalization regarding embryogenetic development and reproduction (De Luna‐Valdez *et al*. 2021).

Although significant efforts have been made to study the DXS family in plants, its functions and evolutionary roots are not fully understood. For example, the evolutionary state and expansion of the DXS family in early land plants, which hold a key position in the conquest of land, are mostly untouched. However, whole genome and large gene duplications, which represent an important trait in plant evolution and subsequent metabolic specialization, have already occurred to a large extent in bryophytes (Hofberger *et al*. 2015; Gao *et al*. 2022). This is exemplified in the large metabolic abundance in the spreading earthmoss *Physcomitrium patens*, which originated in two ancient whole‐genome duplications (Rensing *et al*. 2007) and is also reflected in a comparatively high number of genes that are associated with rate limitation in the isoprenoid pathway (Chen *et al*. 2018). *P. patens* carries four copies of DXS, but these are all found within one clade (DXS1), unlike angiosperms that mainly harbour DXSs from all three or at least two of the clades (De Luna‐Valdez *et al*. 2021).

This relative abundance is in direct contrast to a comparably low level of metabolic complexity in the downstream pathway, *e.g*., measured by the terpenoid diversity. *P. patens* carries only one diterpenoid synthase type, a bifunctional *ent*‐kaurene synthase (PpCPS/KS) (Hayashi *et al*. 2006; Zhan *et al*. 2015), and lacks other non‐essential types. Diterpenes derived from PpCPS/KS are the common gibberellin precursors *ent*‐kaur‐16‐ene and 16‐hydroxy‐*ent*‐kaurene, with proposed functions in regulating protonema differentiation and spore development (Hayashi *et al*. 2010; Vesty *et al*. 2016).

Here, we set out to obtain fundamental insights into the DXS family and terpenoid biosynthesis of *P. patens*. First, we used an *in silico* approach to investigate the DXS family of bryophytes in an evolutionary context in relation to algae and vascular plants. Second, we combined a functional complementation assay and comparative transcriptomic analysis of different tissue types and conditions with co‐expression network analysis to reveal the transcriptional profile and functions. Based on this analysis, we performed a salt stress assay using qRT‐PCR and UHPLC‐QToF‐MS to explore the effects of abiotic stress, more specifically salt stress, on the pathway itself, as well as on the downstream metabolite profile.

## MATERIAL AND METHODS

### Phylogenetic analysis, protein structure analysis, and transcriptome profiling

Phytozome (https://phytozome.jgi.doe.gov/pz/portal.html) and NCBI databases were consulted to extract all DXS amino acid sequences of *P. patens* (Table S1). The sequences were aligned (including transit peptides) with ClustalW in Mega X, and the maximum likelihood bootstrap consensus tree was calculated with default parameters (Figure S1). Signalling peptides were predicted with TargetP 2.0 (https://services.healthtech.dtu.dk/service.php?TargetP‐2.0). For the tissue‐ and condition‐specific expression analysis, selected transcripts per million (TPM) values were retrieved from the publicly accessible EVOREPRO database (https://evorepro.sbs.ntu.edu.sg) (Julca *et al*. 2021) and visualized with ggplot2.

### *Physcomitrium patens* material and growth conditions

*Physcomitrium patens* (Grandsen ecotype, International Moss Stock Center #40001) protonema cultures were grown on solid media at 23 °C with continuous 20–50 W m^−2^ light intensity under sterile conditions. Three weeks prior to treatment, protonema tissue cultures were transferred from solid to liquid phyB media. PhyB media consists of 800 mg Ca(NO_3_)_2_, 250 mg MgSO_4_·7H_2_O, 12.5 mg FeSO_4_·7H_2_0, 0.5 g (NH_4_)2C_4_H_4_O, 10 mL KH_2_PO_4_ buffer (25 g KH_2_PO_4_ per litre and adjusted to pH 6.5 with 4 M KOH), and 0.25 ml trace element solution (110 mg CuSO_4_·5H_2_0, 110 mg ZnSO_4_·7H_2_O, 1228 mg H_3_BO_3_, 778 mg MnCl_2_·4H_2_O, 110 mg CoCl_2_·6H_2_O, 53 mg KI, 50 mg Na_2_MoO_4_·2H_2_O per litre ddH_2_0). For solid medium, 0.7% (w/v) agar was added prior to sterilization by autoclaving at 121 °C.

### Stress treatment

For chemical treatment, 3‐week‐old liquid protonema cultures of *P. patens* were supplemented with respective chemical concentrations (*e.g*., 150 mM NaCl, 15 μM ABA, 400 μM MeJA, 400 μM MeSA). For the desiccation stress experiment, 3‐week‐old protonema were transferred to empty plates and harvested at indicated time points. For all stress treatments, at least three independent biological replicates were grown in a randomized basis.

### RNA extraction and expression analysis using qRT‐PCR

Protonema were harvested from liquid cultures of *P. patens* and immediately frozen in liquid N_2_ at selected time points. Then, RNA was extracted with the Spectrum Plant Total RNA Kit (Sigma Aldrich). Triplicates of total RNA (1 μg) from both untreated and treated protonema were digested with DNAse I (Sigma‐Aldrich) and reverse‐transcribed into cDNA by iScript*—*cDNA Synthesis Kit (BioRad) following the manufacturer's protocol. Quantitative Real‐Time PCR was run on Biorad CFX Connect using gene‐specific primers and SYBR™ Green PCR Master Mix (Thermo Fisher) in 96‐well plates. The thermocycler was programmed to run for 3 min at 95 °C, followed by 40 cycles of 10 s at 95 °C and 30 s at 60 °C. Three identically treated replicas of each RNA sample were analysed to account for biological variation. Each replica was run twice in the thermocycler to minimize technical variation. Characterization of individual candidate genes was acquired simultaneously with the housekeeping gene in the same runs. Notably, several previously described housekeeping candidates have been evaluated (*beta‐tubulin, elongation factor 1 α* (*EFIA*, *E3*) (Le Bail *et al*. 2013), but the highest consistency under stress was obtained from *Actin*. Thus, *Actin* was used for normalization and the expression levels of all genes were derived from the 2−DDCT method. The qPCR primer sequences can be found in Table S2.

### Functional complementation

The ORFs of *DXS1A*, *DXS1B*, and *DXS1C* were synthesized by Twist Bioscience, while *DXS1D* was previously cloned. The complementation assay was carried out as previously described (Cordoba *et al*. 2011) with a few modifications. The distinct *PpDXSs* sequences were incorporated into the pUC19 plasmid for transformation. All constructs were checked by sequencing. Transformed EcAB4‐2 strain with an empty vector was used as a negative or positive control, depending on mevalonate (MVA) supplementation. Transformants were selected on Luria–Bertani (LB) broth plates with 1 mM MVA, supplemented with kanamycin (25 μg·ml^−1^), chloramphenicol (17 μg·ml^−1^), and ampicillin (100 μg·ml^−1^). Then, three colonies per construct were grown on LB plates supplemented with antibiotics and 0.2 mM IPTG, but no MVA. Single colonies were gently touched with a pipette tip and diluted in 20 μl of sterile Milli‐Q water. Then, 5 μl of each bacterial suspension was added to 195 μl LB broth supplemented with the appropriate antibiotics and IPTG, and with or without MVA for controls. The 96‐well plate was incubated at 37 °C for 25 h at 120 rpm. The bacterial growth was measured using the SpectraMax i3x microplate reader (Molecular Devices, San Jose, CA, USA), configured for endpoint absorbance measurements at a wavelength of 600 nm (OD600). Measurements were taken at 0, 5, and 25 h.

### Relative quantification of metabolites

#### Metabolite extraction

The metabolite extraction and analysis were adapted from previous work (Fu *et al*. 2012) with some modifications. Briefly, fresh *P. patens* from liquid media were harvested and freeze‐dried for 48 h in the dark and stored at −80 °C until extraction. Prior to extraction, freeze‐dried moss material was ground in liquid nitrogen. The extraction was performed in three technical replicates. Approximately 20 mg freeze‐dried moss was weighed directly into a glass tube on an analytical balance, and the precise weight of the moss material noted. After which 3 ml ethanol:hexane (2:1, v/v) containing 0.1% (w/v) butylated hydroxytoluene (BHT) were added to the glass tube, and the mixture ultrasonicated for 20 min in the dark. Subsequently, 2 ml H_2_O and 4 ml hexane were added to the glass tube. The mixture was vortexed thoroughly and centrifuged at 1,000×*g* for 5 min at 4 °C. The 4 ml of hexane (in the upper layer) were transferred to a new glass tube. Another 4 ml of hexane were added twice for the purpose of washing. All hexane mixtures were combined and evaporated under nitrogen at room temperature. The extract was reconstituted in 500 μl methyl tertiary butyl ether (MTBE) + acetonitrile (ACN) (75:25, v:v).

#### Liquid chromatography–mass spectrometry

The UHPLC‐QToF‐MS analysis was conducted on an Agilent Infinity 1290 UHPLC system (Agilent Technologies, Santa Clara, CA, USA) coupled with Agilent 6545 QTOF MS with Dual Jet Stream ESI source and a Diode‐Array Detection (DAD) detector. Mobile phase A comprised H_2_O with 10 mM ammonium acetate, and mobile phase B comprised ACN/MeOH/MTBE (70/20/10, v/v/v). LC separation was performed on an ACQUITY UPLC HSS T3 column (100 Å, 1.8 μm, 2.1 mm × 150 mm) with the following linear gradient: 65% mobile phase B at 0 min, increased to 100% B at 12 min, and kept at 100% B for 4 min, then back to 65% B between 16 and 20 min. Column temperature was kept at 45 °C. The flow rate was 0.5 ml·min^−1^, and the injection volume was 2 μl. DAD detection was set to 450 nM. Mass spectrometry (MS) data were recorded in positive ionization mode (ESI+). The mass range was 100–1700 Da for MS scan and 30–1700 Da for MS/MS scan. The MS/MS spectrum was recorded with fixed collision energies of 10, 20, and 40 eV.

#### Data analysis

The MS‐DIAL program (v 4.42) (Tada *et al*. 2020) was used for processing the LC–MS data. Import parameters were: Retention time start, 0 min; Retention time end, 15 min; Mass range start, 100 Da; Mass range end, 1700 Da; MS2 mass range start, 100 Da; MS2 mass range end, 1700 Da; MS1 mass tolerance, 0.02 Da; MS2 mass tolerance, 0.025 Da; Maximum charged number, 2; Minimum peak height, 300; Retention time tolerance for alignment, 0.5 min; MS1 tolerance for alignment, 0.015 Da; Sample max/blank average, 5; Adducts, [M + H]^+^, [M + NH_4_]^+^, [M + Na]^+^, [M + K]^+^, [M + H‐H_2_O]^+^, [M + H‐2H_2_O]^+^. The LipidBlast *in silico* database was used for annotation of lipid metabolites, and the metabolomic MS/MS database was used for identification of pigments. The abundance (integrated peak area) of each metabolite in a biological sample was normalized by the dry weight used for extraction. The normalized results, together with the retention times and adduct types, can be found in Table S3.

Based on the raw data analysis, the relative content of identified metabolites between treatment and control at the respective time points was calculated based on the abundance using the Welch test. Then, adjusted *P*‐values were calculated using the Benjamini‐Hochberg false discovery rate method, and metabolites were considered as significantly changed if *P* < 0.05 (Table S4). The results were visualized with the omu package (Tiffany & Bäumler 2019) and ggplot2.

### Overrepresentation analysis of co‐expression networks

The most significant hits for co‐expression in *P. patens* were retrieved from the Phytozome database, which considers the expression in different strains, under different conditions, and in different tissue types (Table S5). Gene ontology in the co‐expression networks was calculated with the BiNGO plugin in Cytoscape (Shannon *et al*. 2003; Maere *et al*. 2005). The binominal test with Benjamini–Hochberg false discovery rate and statistical correction was applied (Table S6). Most significant non‐redundant hits were visualized in ggplot2.

## RESULTS

### The PpDXS family belongs to the conserved phylogenetic DXS 1 clade

**Fig. 1:**
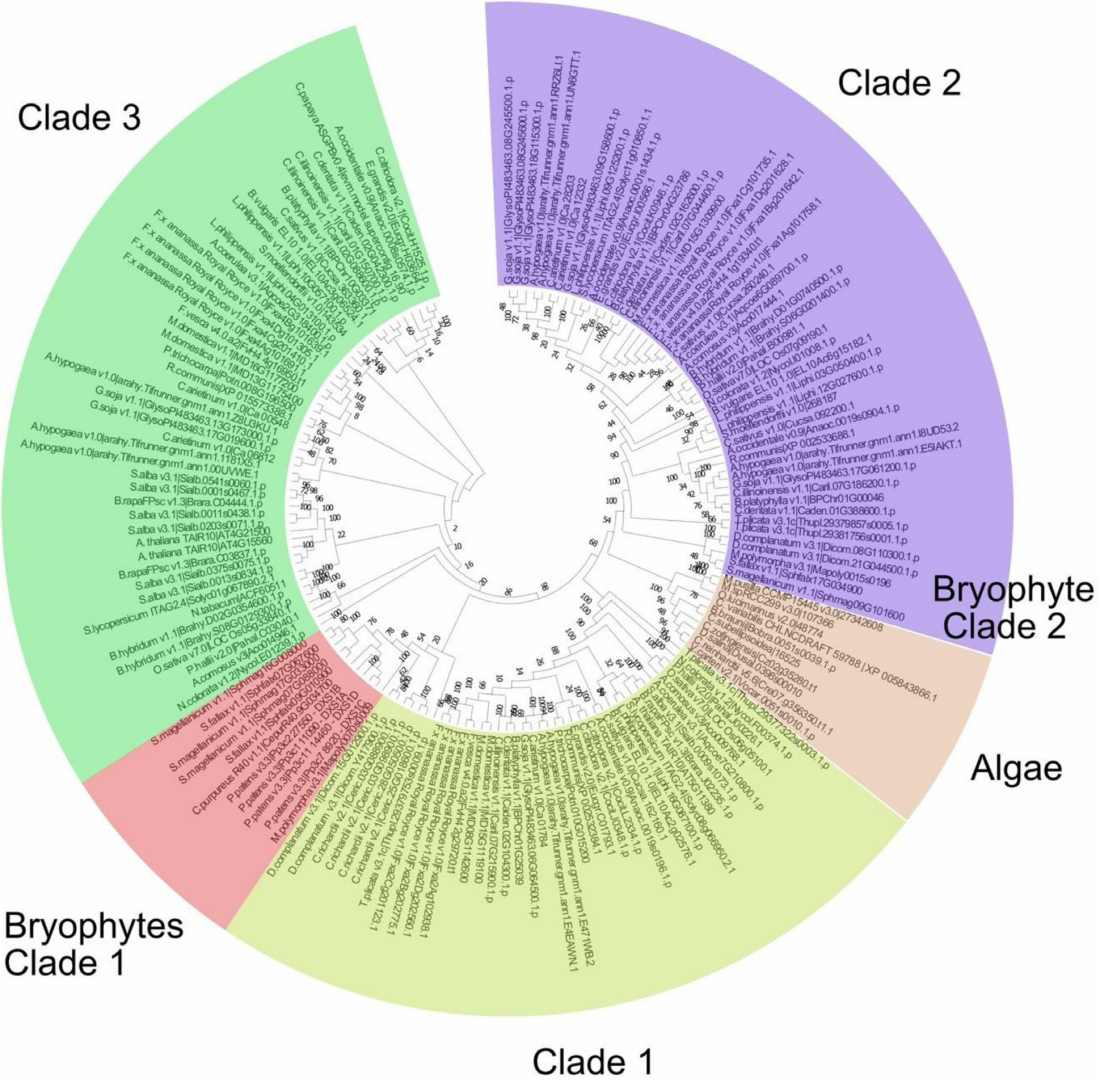
Maximum likelihood bootstrap consensus tree of plant DXS proteins in *P. patens*. The tree was constructed with the maximum‐likelihood method using MEGA X. Phytozome and NCBI accession numbers of DXS amino acid sequences can be found in Table S1. The colours indicate different clades and conserved subtrees. Light green marks DXS clade 1, pink is clade 2, green is clade 3, red is clade 1‐like DXS from bryophytes and light brown DXS from algae.

A total of 162 DXS amino acid sequences, including those of all available bryophyte members, were retrieved from the Phytozome database, and a phylogenetic tree was constructed to evaluate the evolutionary distance in the DXS family between algae, mosses, and vascular plants (Fig. 1). The analysis shows the majority of bryophyte DXS copies as an integral part of the DXS1 clade, with a significant distance from algae. Moreover, this analysis indicates that, in contrast to algae, the DXS family has already expanded structurally in mosses and liverworts. The copies from some members of the Magnopsida and Spagnopsida family divide phylogenetically between the DXS1 and DXS2 clades. Despite the high copy number in *P. patens*, all DXS copies shown in Fig. 1 appear to be limited to the DXS1 clade. But with limited genomic data available for bryophytes, it is not possible to determine whether mosses in general are different from sphagnum mosses and liverworts.

To study the structural composition, the amino acid sequences of *P. patens* were aligned together with DXS sequences from all other available bryophytes and the DXS family from *Oryza sativa*, which harbours a DXS copy of each clade (OsDXS1, OsDXS2, and OsDXS3) (Figure S2). This analysis highlights conservation in the TPP‐ and GAP‐binding domains in all PpDXS copies, together with OsDXS1 and all DXS copies from the bryophyte group, which cluster as DXS1 (Xiang *et al*. 2007). SpmDXS2, SfDXS2, and MpDXS2, which all cluster together with the DXS2 clade, form structural similarities with OsDXS2, and show lower conservation in and around the catalytical centers.

Overall, the four PpDXS copies had a sequence homology of 87%. The highest variation in the sequence can be observed in the N‐terminal region. Plastid targeting was predicted for all four copies (Figure S2). The phylogenetic analysis show that all *Pp*DXS copies are of the DXS1‐type, and thus the four copies are referred to as *Pp*DXS1A (Pp3c1_11090), *Pp*DXS1B (Pp3c2_27550), *Pp*DXS1C (Pp3c7_8920), and *Pp*DXS1D (Pp3c11_14460) in this study.

### All four *PpDXS* copies encode functional enzymes

**Fig. 2:**
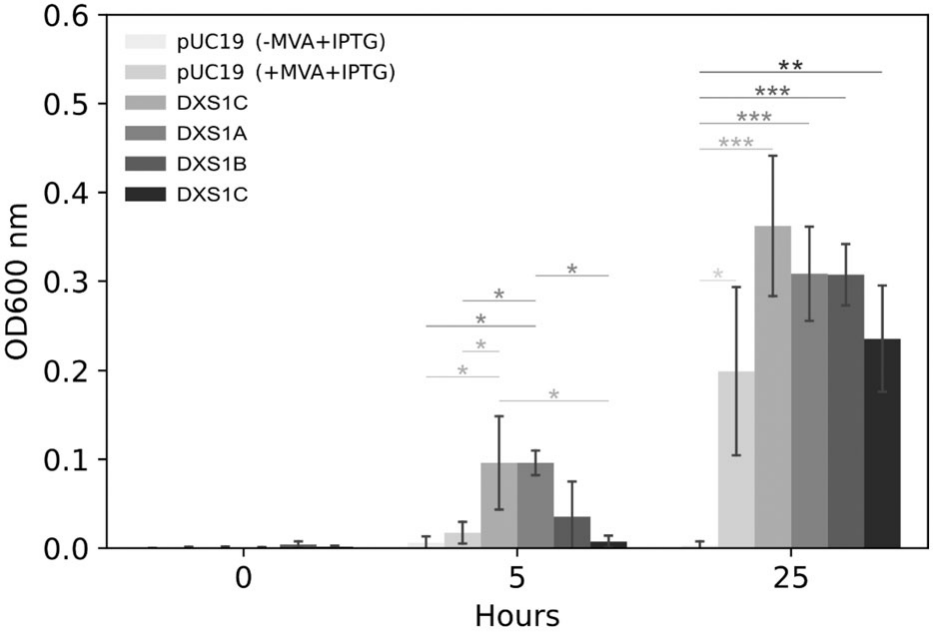
Functional complementation of the PpDXS proteins in *E. coli*. The *E. coli* strain EcAB4‐2, defective in DXS activity, was complemented with each of the four PpDXS genes and grown in the absence of mevalonate. Growing capacity was followed by optical absorbance at 600 nm at 5 and 25 h. EcAB4‐2 under exogenous mevalonate supply was used as a positive control. No growth was observed at these times in the empty vector, used as a negative control. The means ± SE of three cultures (*n* = 3) are shown. Asterisks above bars represent level of significance (* < 0.05, ** < 0.005, *** < 0.0005) as determined by paired *t*‐test.

A functional complementation assay was conducted to determine whether the enzyme activity was retained in all four DXS copies. The deletion of specific MEP pathway genes in *E. coli* strains that have the synthetic MVA+ operon leads to a lethal phenotype, which can be saved by externally providing MVA (Campos *et al*. 2001). For our purpose, the DXS‐deficient *E. coli* strain EcAB4‐2 was used (Sauret‐Güeto *et al*. 2006). This strain cannot grow without exogenous mevalonate unless a functional DXS protein is introduced. The defective strain was transformed with the ORFs of the four DXS copies under control of an inducible bacterial promoter. Transformants harbouring DXS1A and DXS1C showed significant growth after 5 h (Fig. 2). After 25 h, transformants harbouring DXS1B and DXS1D also showed significant growth, similar to the positive control that was supplemented with exogenous mevalonate. In summary, this assay confirms that functional activity was retained in all four copies.

### Tissue‐, light‐ and stress‐specific modulation of gene expression in the PpDXS family

**Fig. 3:**
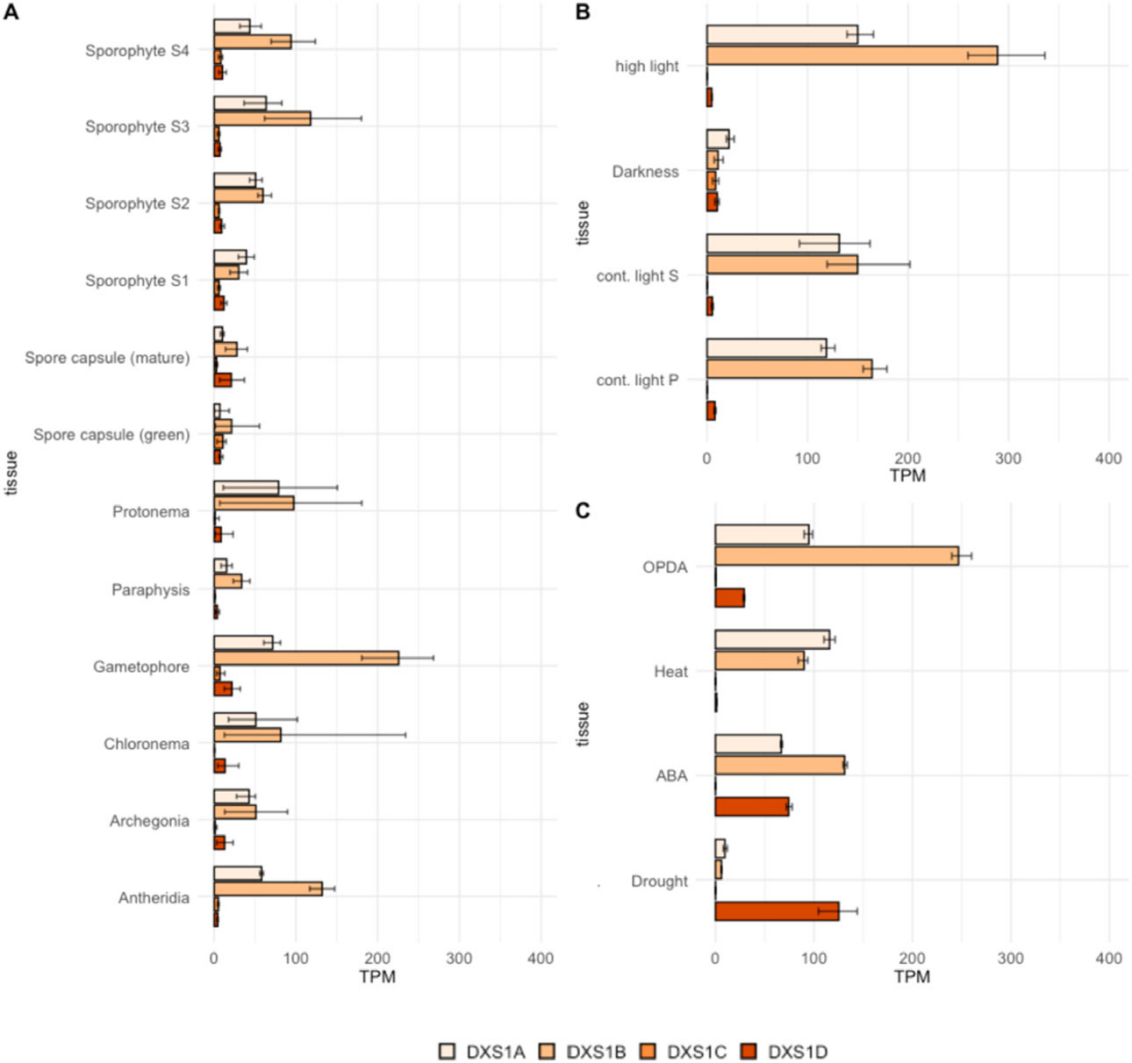
Transcriptional profiling in the DXS family of *P. patens* across different development stages and conditions. Absolute expression (TPM) values were retrieved from the EVOREPRO database and plotted with ggplot2. (A) *PpDXS* expression across different development stages. (B) *PpDXS* expression in absence or presence of light, in protonema/gametophores, and spores (C) *PpDXS* expression under the exposure to stress. The drought stress was conducted with gametophore cultures, whereas a combination of protonema and gametophores was used for the heat and OPDA treatment. Protonema was utilized for the ABA treatment. More information on the experimental conditions can be found in the EVOREPRO database and in the *Physcomitrium* gene atlas (Perroud *et al*. 2018).

To gain insights into the transcriptional patterns within the *PpDXS* family, a selection of gene expression data were retrieved from the EVOREPRO database (https://evorepro.sbs.ntu.edu.sg) (Julca *et al*. 2021). Different tissue types and developmental stages were compared and visualized in ggplot2 (Fig. 3A). *PpDXS1A/B* were ubiquitously expressed throughout all development stages, with elevated expression levels in photosynthetically active tissues like chloronema, protonema, and gametophores. Under the influence of light, the expression in leafy tissue and germinating spores was further increased, whereas the expression was completely shut down in the absence of light (Fig. 3B). In contrast, *PpDXS1C/D* showed little to no response across different tissue types and in response to light stimulus (Fig. 3A,B). Interestingly, *PpDXS1D* exhibited a strong increase after abscisic acid (ABA) application and during drought stress in protonema and gametophores, respectively, accompanied by a simultaneous decrease in expression of *PpDXS1A/B* (Fig. 3C). Moreover, a moderate increase could be monitored in protonema and gametophores treated with the MeJA precursor, 12‐oxo phytodienoic acid (OPDA).

To investigate stress responsiveness of *PpDXS1D* in detail, the effects of different abiotic (salinity, ABA, drought) and biotic (MeJ, MeSA) conditions on the terpenoid precursor pathway were monitored in a qRT‐PCR assay. To study the overall impact of stress on the terpenoid precursor pathway, we extended the assay to other major and minor rate‐limiting steps, such as 1‐deoxy‐D‐xylulose 5‐phosphate reductoisomerase (*DXR*), isopentenyl‐diphosphate delta‐isomerase (*IDI*), hydroxy‐3‐methylglutaryl reductase (*HMGR*), and 3‐hydroxy‐3‐methylglutaryl coenzyme A synthase (*HMGS*). As further controls, expression of the sodium pump ATPase enaI, dehydrin, 12‐oxophytodienoic acid reductase 3 (*OPR3*) and phenylalanine ammonia‐lyase 1 (*PAL1*) were measured to validate the transcriptional response to 150 mM sodium chloride (NaCl), 15 μM ABA, drought, 400 μM MeJa, and 400 μM MeSa, respectively (Figures S2 and S3A–E) (Fraile‐Escanciano *et al*. 2009; Fesenko *et al*. 2019).

Similar to the transcriptomic data, we found that ABA, drought, and NaCl, triggered a strong increase in *PpDXS1D* expression within 24 h following the treatment, with application of 15 μM ABA and 150 mM NaCl sparking an imminent response with a 10–15‐fold increase in *PpDXS1D* (Fig. 3A, B). Under the effect of desiccation, there was a progressive increase in *PpDXS1D* (Fig. 3C). Notably, in the transcriptomic data, the upregulation was accompanied by a downregulation of *PpDXS1B*, and also of *PpDXS1A* (Fig. 3A–C, F–H) under drought stress, as well as an increase in *PpHMGR1*.

**Fig. 4:**
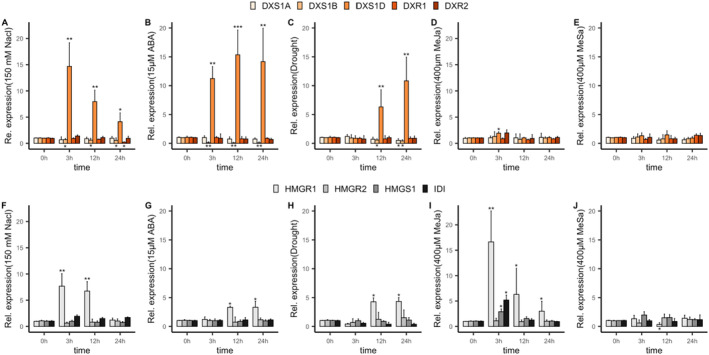
Relative expression of major and minor rate‐limiting steps in the terpenoid precursor pathway under the influence of stress in *P. patens*, based on qRT‐PCR analysis. *PpActin* was used as a housekeeping gene. (A–E) Relative expression of MEP pathway genes under 150 mM NaCl, 15 μM ABA, Drought, 400 μM MeJa, and 400 μM MeSa, respectively. (F–J) Expression of MVA pathway genes under 150 mM NaCl, 15 μM ABA, Drought, 400 μM MeJa, and 400 μM MeSa, respectively. Data are presented as the mean ± SE of three replicates. 0 h is control with no treatment. Asterisks above or below bars represent level of significance (* < 0.05, ** < 0.005, *** < 0.0005) as determined by paired *t‐*test.

Next, we applied MeJA and MeSA to study the response of *P. patens* to biotic stress. We found little effect on gene expression in either pathway (Fig. 4D, E, I, J). Notably, MeJa and MeSa concentrations, ranging from 50 μM to 400 μM, were considered in this study, but concentrations below 400 μM triggered little to no response (Figure S2, Fig. 3F–H) as reported earlier (Fesenko *et al*. 2019). A slight increase in *PpDXS1D* was monitored in response to MeJa at 3 h (Fig. 4D), but considering its naturally low expression profile, the significance is questionable. In contrast, the mevalonate pathway showed a significant response to 400 μM MeJa (Fig. 4I).

Taking the high concentration of MeJa into consideration, a non‐specific response cannot be excluded. Comparable concentrations of MeSa triggered a decrease in Pp*HMGR1* expression but had no further impact on mevalonate and MEP pathways (Fig. 4E, J). From the results presented here, it can be concluded that *DXS*, and potentially the MEP pathway as a whole, do not show a significant response to MeJa and MeSa. In contrast, a moderate response to the direct MeJa‐precursor OPDA was notable in the transcriptome data (Fig. 4C).

### Co‐expression networks of *PpDXS* copies indicate activity in different function clusters

**Fig. 5:**
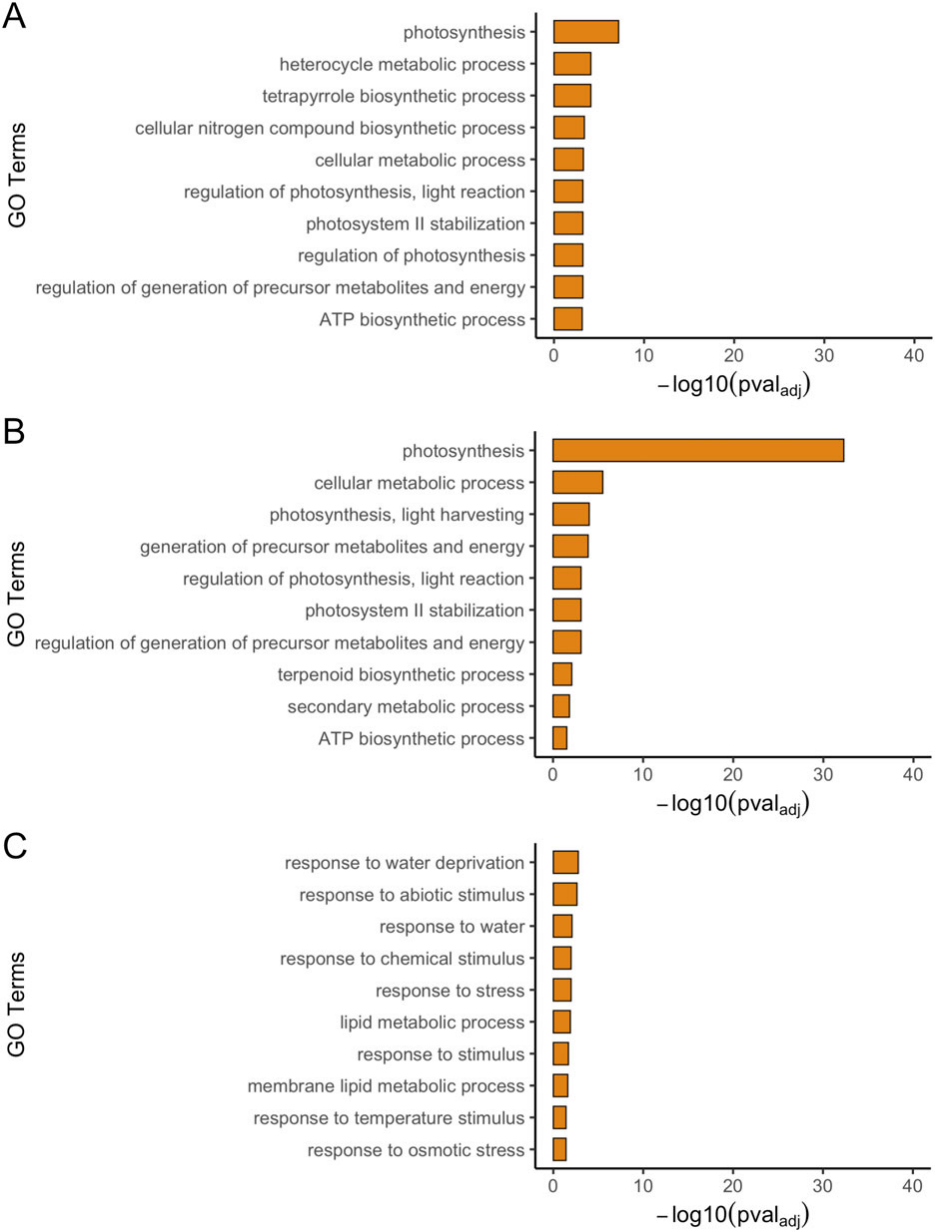
Gene ontology (GO) enrichment analysis of biological processes in the gene co‐expression networks of *PpDXS1A* (A) *PpDXS1B* (B) and *PpDXS1D* (C) in *P. patens*. The GO enrichment analysis in the co‐expression networks was retrieved using Cytoscape BiNGO. The 10 most significantly enriched non‐redundant processes are visualized here. A detailed overview of all enriched processes can be found in Table S6.

To make predictions on potential functions, the gene composition in the co‐expression network of the *PpDXS* family was analysed for *P. patens*. The utilization of co‐expression network analysis to predict gene functions is gaining traction (Gillis & Pavlidis 2012; Wang *et al*. 2017) and was also employed recently to elucidate putative functional interactions in the DXS family of *A. thaliana* (De Luna‐Valdez *et al*. 2021). The co‐expression networks for *PpDXS1A*—*PpDXS1D* were retrieved from the Phytozome database (Table S5), and a detailed list of all enriched GO terms can be found in Table S6. The overrepresentation analysis of biological processes in the respective networks was conducted with the BiNGO plugin in Cytoscape (Fig. 5), with default parameters and multiple testing corrections using FDR correction. A selection of the 10 most significantly enriched non‐redundant terms was visualized in ggplot2 (Fig. 5). This analysis shows that *PpDXS1A/B* are co‐expressed with processes including photosynthesis and photosynthesis‐related processes, such as the regulation of photosynthesis, protection of the photosystem II, and biosynthesis of chlorophylls. In other plants, these processes are associated with clade 1 DXS. Strikingly, the set of genes co‐expressed with *PpDXS1A* and *PpDXS1B*, respectively, shares an overlap of approximately 16% (7 genes; Table S5). In terms of gene‐specific function, both sets of genes reach an overlap of at least 37% (Table S6). These are mostly photosynthesis‐associated processes.

Additionally, the *PpDXS1B* network shows enrichment of genes concomitant to isoprenoid biosynthesis, such as *DXR* and *lycopene cyclase*. In contrast, the *PpDXS1D* network illustrates enrichment of stress response and processes that could potentially be linked to stress response, such as lipid and membrane lipid biosynthesis. Several genes related to the glycerophosphocholine metabolism, including several phospholipase D genes and phosphatidylcholine sterol O‐acyltransferases, are present in this network. A network for *PpDXS1C* could not be established because of a lack of statistical significance, probably related to low expression level of the gene.

### Increased metabolic flux towards isoprenoids under salt stress

Finally, a metabolomic analysis was performed to study the impacts of salt stress on the metabolite profile in the downstream pathway of *P. patens*. Liquid protonema cultures were grown in phyB media, supplemented with either 0 mM or 150 mM NaCl, and sampled after 12 h and 7 days, respectively. For the analysis, a previously described extraction‐ and mass spectrometry‐based protocol for the quantification of chlorophylls and carotenoids was adopted (Fu *et al*. 2012). Using this extraction method, we detected eight types of pigment but also 174 lipid metabolites using MS‐DIAL. The lipid metabolites were classified by their functional headgroups on the glycerol backbones. In the dataset, short (12 h)‐ as well as long (7 days)‐term response was compared (Table S3). According to the PCA analysis, 41.56% of the changes were related to the treatment (Figure S4). Alteration in the profile of 130 lipids and pigments became evident in the short term, and 100 were significantly altered after 7 days (Table S4). One‐third of the compounds were involved exclusively in a short‐term response.

**Fig. 6:**
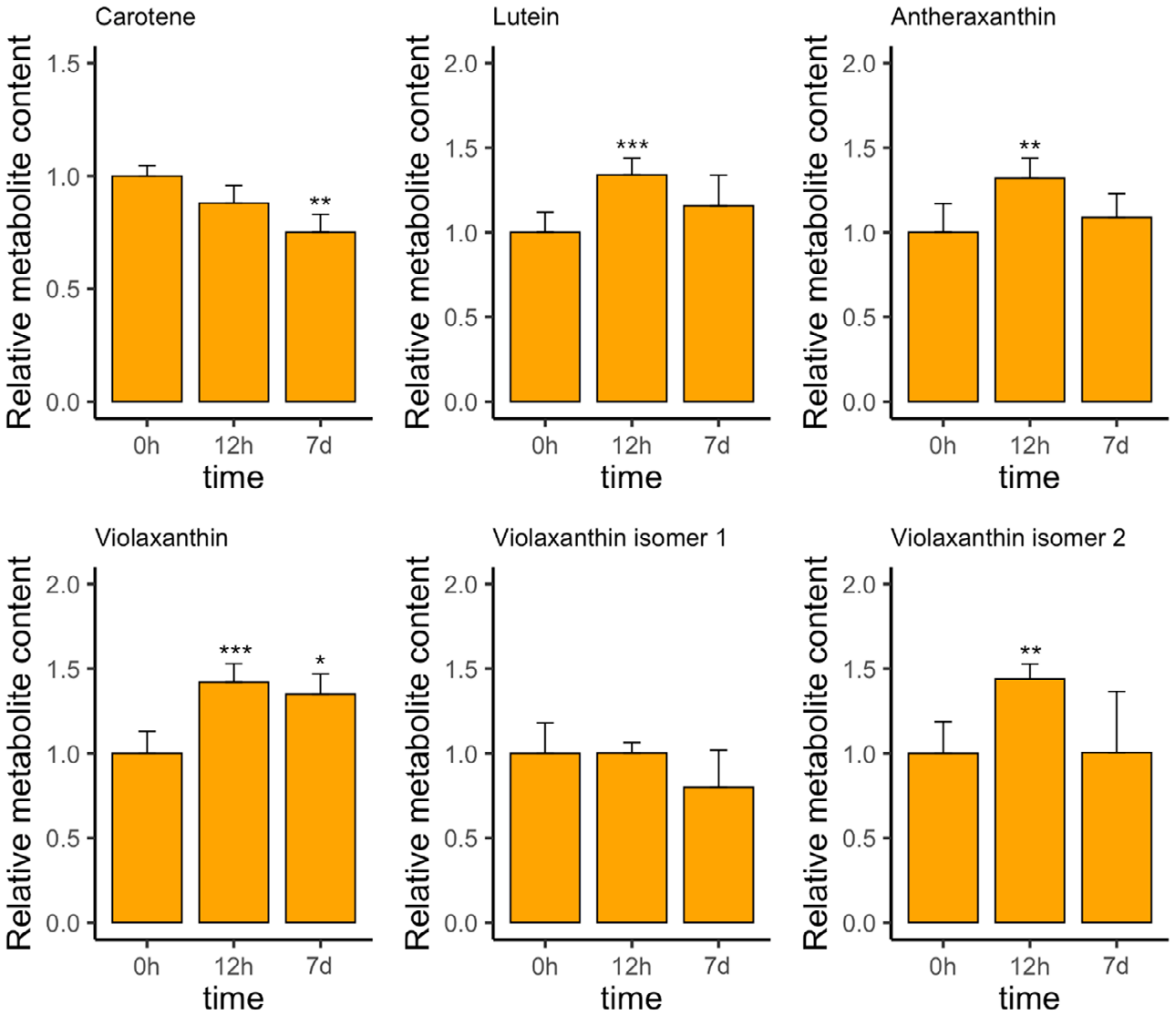
Relative quantities of selected metabolites of *P. patens* in 150 mM NaCl, measured by UHPLC‐QToF‐MS analysis. The differences between salt‐treated and control samples were calculated with the Welch *t*‐test. Then, adjusted *P*‐values were calculated using the Benjamini‐Hochberg false discovery rate method, and metabolites were considered significantly changed if *P* < 0.05. Asterisks above bars represent level of significance (* < 0.05, ** < 0.005, *** < 0.0005).

Significant changes in the abundance of several isoprenoids became evident (Fig. 6). Four out of seven identified isoprenoids showed significantly enhanced, up to around 60% 12 h after the treatment. Amongst these were five xanthophylls (lutein, antheraxanthin, violaxanthin, and two violaxanthin isomers; Fig. 6). After 7 days, violaxanthin was still around 40% upregulated, and lutein around 20%, but other carotenoids either did not show differences or were downregulated (beta‐carotene, Fig. 6).

## DISCUSSION

In the present study, we explored transcriptional and functional characteristics in the terpenoid biosynthesis and the DXS family of *P. patens*, and correlations with abiotic stress response. First, we used an *in‐silico* analysis to investigate structural composition in the DXS family. Second, we determined the level of functional activity in this family. Subsequently, assessing the gene expression and co‐expression networks allowed us to explore the transcriptional landscape of the specific DXS genes. Our findings show that, in contrast to other bryophytes and vascular plants, the PpDXS family did not expand phylogenetically and clusters within the DXS1 clade. Interestingly, our functional complementation assay showed that all copies retained their functional activity. Nevertheless, we found light‐, tissue‐ and stress‐specific divergence in the transcriptional dynamics, with *PpDXS1D* being mainly stress‐responsive. Additionally, we found that the transcriptional response of *PpDXS1D* coincidences with enrichment of stress‐responsive genes in its co‐expression network and the accumulation of stress‐responsive metabolites from the downstream terpenoid pathway in salt stress.

Based on previous phylogenetic analysis, the *P. patens* DXS family appears either as part of a bryophyte‐specific subtree that shares the same ancestral branch with the DXS3 clade (Zeng & Dehesh 2021), or as part of a bryophyte‐specific subtree integral to the DXS1 clade (De Luna‐Valdez *et al*. 2021). These studies applied the maximum likelihood algorithm with different parameters to investigate the phylogeny: the first study investigated the evolutionary origin by emphasizing differences between plants and microbial ancestors, and the second focused on division in the plant kingdom while excluding transit peptides, as these account for high variability. In the present study, we analysed the phylogeny of the DXS family using the maximum likelihood model with an emphasis on bryophytes and their interrelation with algae and vascular plants, and we retained the transit peptides. Our findings overlap with De Luna‐Valdez *et al*. (2021), suggesting a common ancestral branch for the bryophyte subgroup with the DXS1‐type. Despite the discrepancies in the relationship and structure of the different DXS groups between studies, they all agree that an expansion of the DXS family occurred already in both liverworts and mosses. However, it seems that not all mosses followed this expansion (*e.g., P. patens*, *C. purpureus*). As genomic resources and full genome annotations in liverworts are still limited, it is unclear whether the DXS family has already expanded in liverworts as much as it has in vascular plants (Hofberger *et al*. 2015). These knowledge gaps provide interesting avenues to explore the genetic and metabolic diversity in mosses. Although the clustering of PpDXSs with DXS1 proteins and their preservation of DXS activity serve as evidence that they are genuine DXS1 counterparts, distinct from DXS3 proteins, which lack DXS activity.

Our transcriptional analysis revealed expression of at least three *DXS* copies. The expression of these genes was significantly different across tissue types, development stages, and various stimuli. For *PpDXS1A/B*, we observed ubiquitous gene expression across different development stages. Both are light‐responsive and show co‐expression with various photosynthetic processes. In other plants like *A. thaliana*, a role in photosynthesis is characteristic of the DXS1 clade (Walter *et al*. 2002; Kim *et al*. 2005; Zhang *et al*. 2009, 2020). Moreover, the high similarity in gene and protein structure, overlapping expression patterns, and the comparative co‐expression networks, suggest genetic redundancy between the two copies. In contrast, *PpDXS1C/D* showed significantly different gene expression patterns compared to *PpDXS1A/B*. Both *PpDXS1C* and *PpDXS1D* showed remarkably low expression throughout the life cycle. Nonetheless, *PpDXS1D* exhibited a strong response to salt stress. This is reflected in the co‐expression network, which demonstrates an enrichment of stress responses (Fig. 5). In our qRT‐PCR assay, we found that the *PpDXS1D* expression has a significant negative correlation with *PpDXS1B* under abiotic stresses (salt, ABA and drought), as well as with *PpDXS1A* under drought stress. With regard to salt stress in particular, it is possible that *PpDXS1D* upregulation may be able to compensate for reduced gene expression in *PpDXS1A/B*. High gene copy numbers are a distinct trait of the *P. patens* genome (Rensing *et al*. 2007). Growing evidence suggests that the retention of significant amounts of stress‐related genes in the genome can help organisms cope with terrestrial stressors concomitant with the conquering of land (Gao *et al*. 2022) In this context, the stress‐specific compensation of transcriptional activity by *PpDXS1D* may ensure sufficient supplies of precursors for downstream metabolites, some of which play a role in response to stress (*e.g*., ROS scavenging by carotenoids) (Latowski *et al*. 2011; Uarrota *et al*. 2018). In summary, the genetic redundancy in the PpDXS family may benefit the stress‐resistance in *P. patens*.

In plants, carotenoids play an important role in stress response, *e.g*., ROS scavenging (Das & Roychoudhury 2014; You & Chan 2015). The literature shows that carotenoid content is enhanced by salt stress (Sankari *et al*. 2019; Fal *et al*. 2022), and is influenced by gene expression of *DXS* (Morris 2006; Yang *et al*. 2012). Also, the concept of DXS‐mediated flux control in the isoprenoid pathway supply has been revealed in *A. thaliana* (Wright *et al*. 2014). In our study, most of the affected carotenoids were xanthophylls, with increased levels at 12 h. These play an important role in defence against ROS generated by oxidative stress and function as a precursor in the biosynthesis of ABA (Latowski *et al*. 2011). Since the content of most carotenoids was not significantly elevated at 7 days, our findings suggest carotenoids are involved particularly in the earlier stages of the salt stress response (*e.g*. ROS scavenging). Our results also overlap with previous findings that show carotenoid accumulation after salinity exposure in *P. patens* (Azzabi *et al*. 2012). Besides, the accumulation of isoprenoid‐containing lipid droplets under salt stress in *P. patens* has been reported recently (Ćosić *et al*. 2021).

Additionally, we only observed very small response in the MEP pathway of *P. patens* against biotic stress (Fig. 4). Although a stress response could be observed in the mevalonate pathway to MeJa, it should be noted that a response could only be triggered at high MeJa concentrations. The presence of MeJa and the responsiveness to it remains unclear in *P. patens*, as only its direct precursor OPDA has been detected in mosses (Ponce De León *et al*. 2012). OPDA is known to be involved in a stress response to *Botrytis* infection, but the transcriptomic data suggest no to little response in both the MEP and mevalonate pathways (Fig. 2). Notably, mosses are generally rarely consumed by animals, likely because of their distinct cellular composition (Gerson 1982; Horn *et al*. 2021). Low selection pressure might have supported conservation of the DXS type 1 in *P. patens*.

Overall, our study suggests genetic redundancy in the four PpDXS copies, and highlights strong divergence in the gene expression profile of the *DXS* family in *P. patens*, with *PpDXS1D* transcription in particular underlying stress‐specific regulation. Whether this genetic redundancy is to support the organism's resilience to environmental conditions or to promote metabolic diversity remains to be studied. Complementary to our insights into transcriptional regulation, follow‐up studies should also consider the role of post‐transcriptional and post‐translational modifications.

## AUTHOR CONTRIBUTIONS

A.H., Y.L., and J.D.B. designed the experiments. A.H., Y.L., and F.J.A.R. performed the experiments, analysed the data and wrote the manuscript. H.T.S. and J.D.B. revised the manuscript. All authors contributed to the article and approved the submitted version.

## CONFLICT OF INTEREST

The authors declare no conflicts of interest.

## FUNDING INFORMATION

AH and YL received funding from the European Union's Horizon 2020 research and innovation programme under the Marie Sklodowska‐Curie grant agreement (No. 765115‐MossTech). FJAR was supported by a stipend from Agencia Nacional de Investigacion y Desarollo, Chile. Moreover, this work was supported by FCT (Fundação para a Ciência e a Tecnologia), I.P. through “GREEN‐IT Bioresources for Sustainability” Unit (UIDB/04551/2020, DOI: 10.54499/UIDB/04551/2020; 10.54499/UIDP/04551/2020), and through LS4FUTURE Associated Laboratory (LA/P/0087/2020; DOI: 10.54499/LA/P/0087/2020). JDB received salary support from FCT through grant CEECIND/03345/2018.

## Supporting information


**Table S1.** List of DXS accession numbers.

**Table S2.** List of qRT‐PCR primers.

**Table S3.** Metabolome data from UHPLC‐QToF‐MS.

**Table S4.** Significantly altered metabolites in *P. patens* after 150 mM NaCl treatment at 12 h and 7 days, respectively.

**Table S5.** Co‐expression networks of the different *DXS* copies in *P. patens*.

**Table S6.** Overrepresented GO terms.

**Figure S1.** Phylogenetic relationship amongst DXS proteins of plants.

**Figure S2.** Multiple sequence alignment of the PpDXS family with other DXS from bryophytes and *Oryza sativa*.

**Figure S3.** qPCR time‐series analysis of candidate gene expression in liquid protonema cultures of *P. patens* under stress exposure.

**Figure S4.** PCA analysis of identified compounds in the *P. patens* metabolome with and without salt stress at different time points.

## Data Availability

All data supporting the findings of this study are available within the paper and within its supplementary material published online.
