# Supplementary material for: Transcriptional and functional characterization in the terpenoid precursor pathway of the early land plant Physcomitrium patens
Source: Plant Biol (Stuttg). 2024 Nov 27;27(1):29–39. doi: 10.1111/plb.13741 (PMC11656282; doi:10.1111/plb.13741)
Supplement: Supplementary file 8 — Figure S2. Multiple sequence alignment of the PpDXS family with other DXS from bryophytes and Oryza sativa. [file PLB-27-29-s006.pdf]

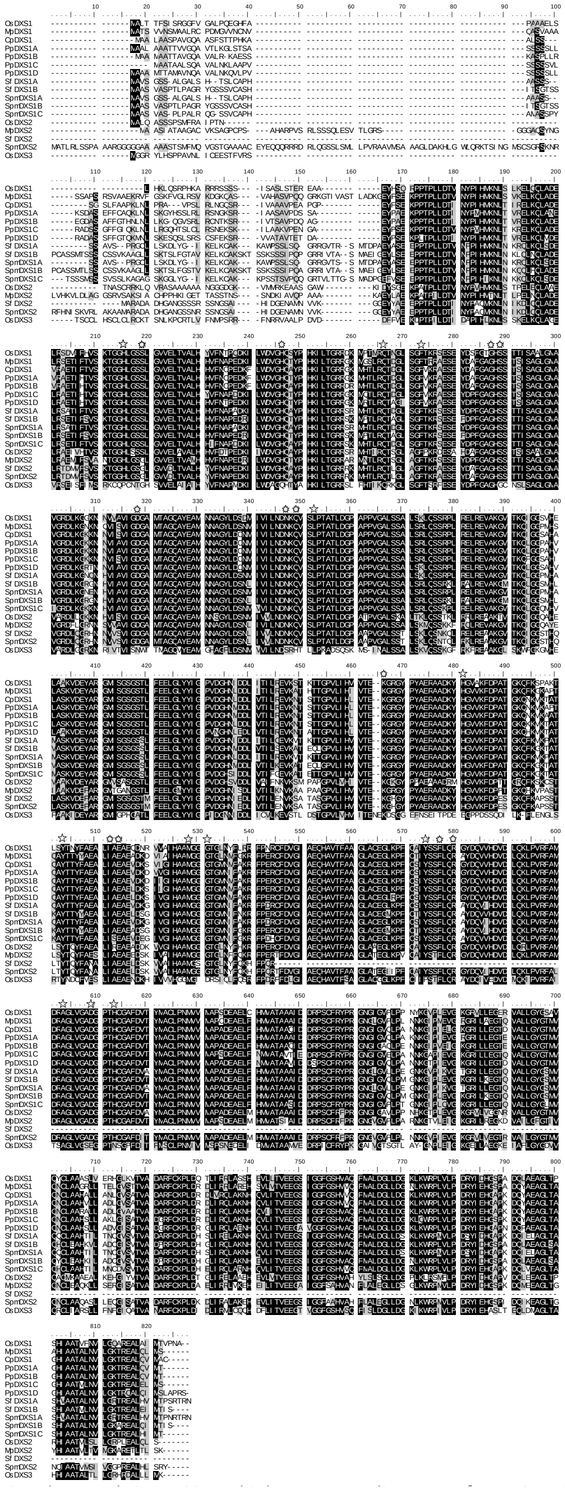

Supplementary figure S2. Multiple sequence alignment of PpDXS amino acid sequences with other DXS sequences from Bryophytes and O. sativa. A detailed list of the accession numbers can be found in supplemental table S1. The predicted chloroplast peptide region for P. patens appears coloured. Solid stars represent the functional residues involved in GAP-binding and hollow stars represent the functional residues involved in TPP-binding.
