# Supplementary material for: Transcriptional and functional characterization in the terpenoid precursor pathway of the early land plant Physcomitrium patens
Source: Plant Biol (Stuttg). 2024 Nov 27;27(1):29–39. doi: 10.1111/plb.13741 (PMC11656282; doi:10.1111/plb.13741)
Supplement: Supplementary file 9 — Figure S3. qPCR time‐series analysis of candidate gene expression in liquid protonema cultures of P. patens under stress exposure. [file PLB-27-29-s007.pdf]

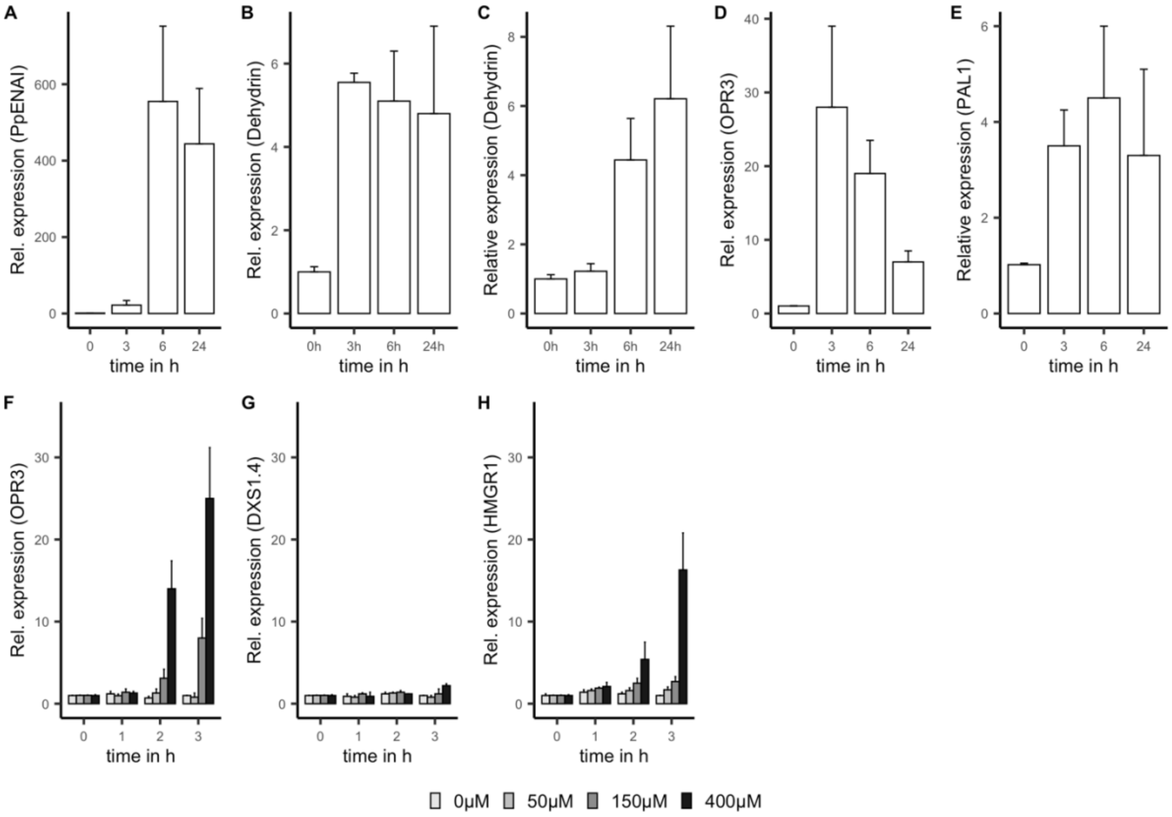

Supplementary figure S3. qRT-PCR time-series analysis of candidate gene expression in liquid protonema cultures under stress exposure. A) Expression of PpENA1 sodium pump in 150mM NaCl at respective time points. B) Expression of dehydrin in 15µM ABA at respective time points. C) Expression of dehydrin under dessication at respective time points. D) Expression of OPR3 in 400µM MeJA at respective time points. E) Expression of PAL1 in 400µM MeSA at respective time points. F) – H) Expression of OPR3, DXS1D and HMGR1 at different MeJA concentrations and at diverse time points.
