# Supplementary material for: Transcriptional and functional characterization in the terpenoid precursor pathway of the early land plant Physcomitrium patens
Source: Plant Biol (Stuttg). 2024 Nov 27;27(1):29–39. doi: 10.1111/plb.13741 (PMC11656282; doi:10.1111/plb.13741)
Supplement: Supplementary file 10 — Figure S4. PCA analysis of identified compounds in the P. patens metabolome with and without salt stress at different time points. [file PLB-27-29-s005.pdf]

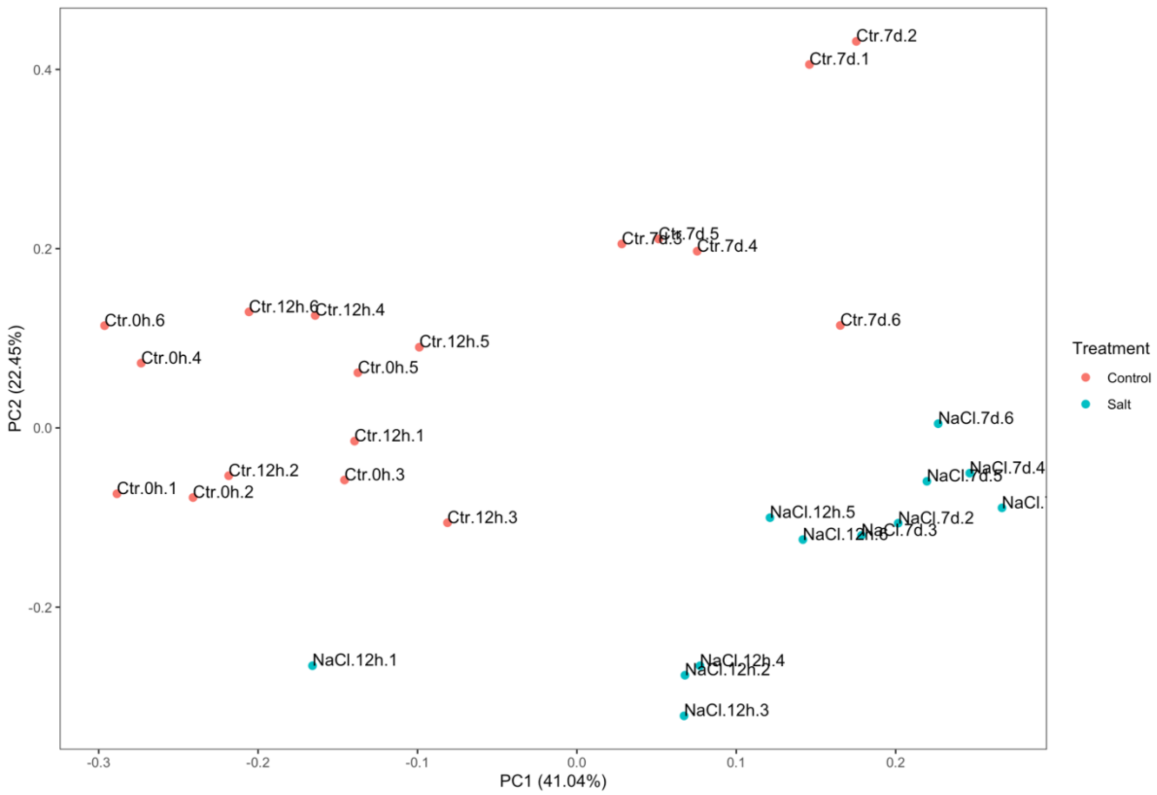

Supplementary figure S4. PCA analysis of identified compounds in the P. patens metabolome with and without salt stress at different time points. Ctr: Control.
