# Supplementary material for: Transcriptional and functional characterization in the terpenoid precursor pathway of the early land plant Physcomitrium patens
Source: Plant Biol (Stuttg). 2024 Nov 27;27(1):29–39. doi: 10.1111/plb.13741 (PMC11656282; doi:10.1111/plb.13741)
Supplement: Supplementary file 7 — Figure S1. Phylogenetic relationship amongst DXS proteins of plants. [file PLB-27-29-s008.pdf]

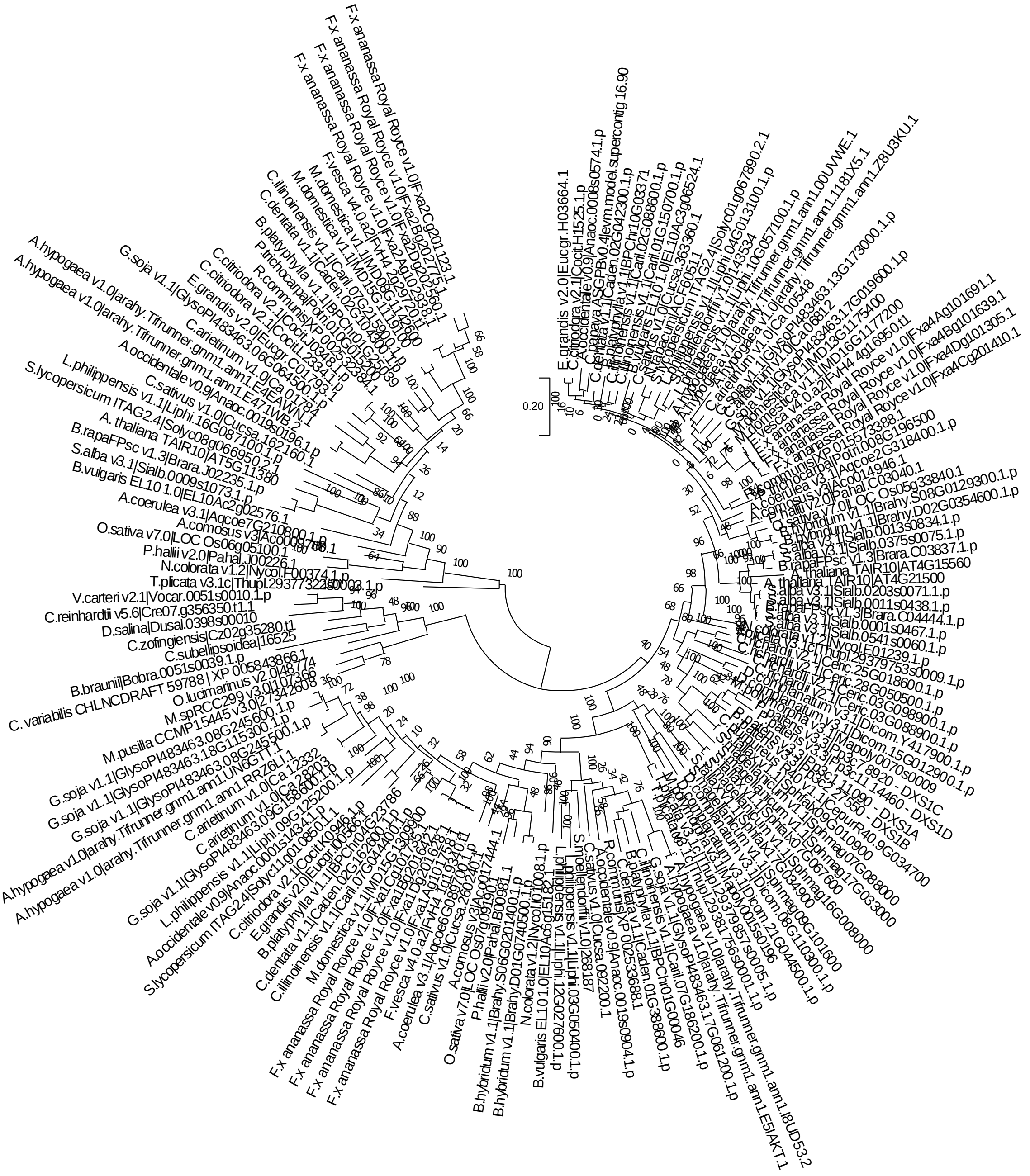

Supplementary figure S1. Phylogenetic relationship amongst DXS proteins of plants. The tree was calculated with the maximum likelihood bootstrap algorithm and is drawn to scale, with branch lengths proportional to evolutionary distance between nodes.
